# Genetic parameters for milk yield in imported Jersey and Jersey-Friesian cows using daily milk records in Sri Lanka

**DOI:** 10.5713/ajas.19.0798

**Published:** 2020-02-25

**Authors:** Amali Malshani Samaraweera, Vinzent Boerner, Hewa Waduge Cyril, Julius van der Werf, Susanne Hermesch

**Affiliations:** 1 Animal Genetics & Breeding Unit, a joint venture between NSW Department of Agriculture and University of New England, University of New England, Armidale 2351, NSW, Australia; 2 Department of Animal Science, Uva Wellassa University, Badulla 90000, Sri Lanka; 3 National Livestock Development Board, Narahenpita 00500, Sri Lanka; 4 School of Environmental and Rural Science, University of New England, Armidale 2351, NSW, Australia

**Keywords:** Dairy Cattle, 305-Day Milk Yield, Daily Milk Yields, Random Regression, Heritability, Tropical Climate

## Abstract

**Objective:**

This study was conducted to estimate genetic parameters for milk yield traits using daily milk yield records from parlour data generated in an intensively managed commercial dairy farm with Jersey and Jersey-Friesian cows in Sri Lanka.

**Methods:**

Genetic parameters were estimated for first and second lactation predicted and realized 305-day milk yield using univariate animal models. Genetic parameters were also estimated for total milk yield for each 30-day intervals of the first lactation using univariate animal models and for daily milk yield using random regression models fitting second-order Legendre polynomials and assuming heterogeneous residual variances. Breeding values for predicted 305-day milk yield were estimated using an animal model.

**Results:**

For the first lactation, the heritability of predicted 305-day milk yield in Jersey cows (0.08±0.03) was higher than that of Jersey-Friesian cows (0.02±0.01). The second lactation heritability estimates were similar to that of first lactation. The repeatability of the daily milk records was 0.28±0.01 and the heritability ranged from 0.002±0.05 to 0.19±0.02 depending on day of milk. Pearson product-moment correlations between the bull estimated breeding values (EBVs) in Australia and bull EBVs in Sri Lanka for 305-day milk yield were 0.39 in Jersey cows and −0.35 in Jersey-Friesian cows.

**Conclusion:**

The heritabilities estimated for milk yield in Jersey and Jersey-Friesian cows in Sri Lanka were low, and were associated with low additive genetic variances for the traits. Sire differences in Australia were not expressed in the tropical low-country of Sri Lanka. Therefore, genetic progress achieved by importing genetic material from Australia can be expected to be slow. This emphasizes the need for a within-country evaluation of bulls to produce locally adapted dairy cows.

## INTRODUCTION

Over the last 15 years, the quantity of imported milk-products into Sri Lanka has doubled (100,000 metric tonnes in 2016) consuming a substantial part of the country’s export revenues through importation of milk products [1]. To reduce the cost of importation of milk products (particularly milk powder) and to meet the local demand through enhancing the local dairy sector, the national government initiated programs for the importation of temperate dairy cattle to Sri Lanka. However, it is well documented that these breeds, which have been intensely selected for milk production, can have decreased performance for fitness and reproduction traits in their native temperate environments [2]. These problems are exacerbated in tropical production environments where cows are challenged by heat, diseases, inadequate feed and water supply [3]. A possible solution to these problems is to develop a locally adapted dairy breed based on a foundation stock of imported temperate dairy cattle.

A precondition for the efficient implementation of breed ing programs is the availability of reliable phenotypic data and pedigrees, enabling estimation of accurate genetic parameters. In developing countries, this precondition is often not met. However, since dairy production in developing countries is in the midst of slow but ongoing change, from an extensive system managed by smallholders with dairy production embedded in a mixed farm operation to larger, more specialized, intensively managed units with modern milking systems [4], the problem of reliable phenotypic data might be overcome by using information automatically recorded in modern milking parlours. Milk yield, milk flow rate, udder conformation are examples of traits automatically recorded depending on the type of the parlour [5]. These traits represent breeding objective traits or may serve as indicator traits. Compared to conventional test-day records, parlour-collected data offers information on each day (and possibly each session) generating large amounts of data. These data allow the cumulative traits (e.g. 305-day yield) to be calculated by summing up the daily parlour records making sophisticated prediction methods [6] developed for test day records, obsolete. In addition, daily records also allow the application of random regression models, which require a minimum data density along the trajectory to produce reliable parameter estimates. However, parlour records caused a much higher fluctuation in daily milk yield compared with the conventional test-day data [7]. Therefore, the data quality of the automatic milking systems may not be sufficient.

This study investigates the feasibility of using automatically recorded milking data in an intensively managed commercial farm for genetic evaluation of imported Jersey and Jersey-Friesian cows in Sri Lanka. Feasibility was assessed by estimating genetic parameters. Genetic parameters for milk yield traits were not estimated in the recent past in Sri Lanka and this is the first study, which utilized daily milk records for genetic evaluation in Sri Lanka. This study presents genetic parameters for 305-day milk yield using univariate animal models and for daily milk yield records using random regression models from data generated in a milking parlour.

## MATERIALS AND METHODS

### Data

Milk yield records were obtained from a single dairy farm in Sri Lanka, which used Jersey and Jersey-Friesian crossbred cows that were imported from Australia as pregnant heifers. The farm is located at 32 meters above sea level (low-country). The local climate is characterized by a distinct wet season, a mean annual temperature of 27 degrees Celsius, an average relative humidity between 70% and 80% and a total annual rainfall from 1,000 to 2,500 mm [8]. Management is characterized by non-grazing and feeding of a total mixed ration produced from cultivated grass and corn, artificial shed cooling, artificial insemination, twice a day milking regime and automatic data recording in a DeLaval milking parlor (Parallel parlour, DeLaval, India).

The first lactation records consisted of 904,437 daily milk records of 2,434 Jersey and Jersey-Friesian crossbred cows, which calved from July 2015 to January 2018. The second lactation records were collected from the same herd and consisted of 456,260 daily milk records of 1,973 Jersey and Jersey-Friesian cows. Calving dates for the second lactation ranged from July 2016 to March 2018. Data editing as outlined below resulted in 663,890 and 391,035 daily records from 2,372 and 1,905 cows from first and second lactations, respectively. The data were recorded from July 2015 to March 2018. Overall, data editing excluded 27% of daily milk records from the first lactation and 14% of daily milk records from the second lactation.

Any milk records taken after 350 days postpartum were removed. A higher percentage of records were removed from the first lactation (15%) than the second lactation (0.5%) as records exceeding 350 days postpartum. Then, records identified as valid by the milk recording system were extracted (11% data loss in first and second lactations). This data edit showed that the minimum milk yield identified by the milking system as a valid daily record was 2 kg. Duplicated days in milk for each cow were removed from analysis. Outliers that differed by more than four standard deviations from the mean within each lactation were excluded from the analysis.

The average number of daughters was 61.3 for sires (range 50 to 78) and 12.6 for maternal grandsires (range 5 to 21) with only 1,314 cows having a known maternal grandsire. No maternal grandsires were used as sires. All sires of the imported cows were Jersey bulls and maternal grandsires were either Jersey (for 521 cows) or Friesian (for 793 cows) bulls. Since the dams of imported cows were all unknown, maternal grandsires were included in the pedigree using dummy dams assuming a unique dam for each offspring. The total number of animals in the pedigree was 3,766 (including dummy dams). Only the animals with records and their parents and grandparents identified were used in the pedigree. The pedigree file with all known relationships consisted of the recorded cows plus the previous two generations. The percentage of Jersey and Friesian contribution in crossbred cows was not known.

### Defining the traits

Altogether four different traits were defined. Two aggregated milk yield traits were defined as predicted 305-day and realized 305-day milk yields. Daily milk yields were used as a longitudinal trait in genetic parameter estimation. In addition, total milk yield for each 30-day intervals across the lactation 1 was used to estimate genetic parameters along the lactation trajectory to provide a comparison with the random regression analysis. Trait definitions for two aggregated milk yield traits are described below.

#### Predicted 305-day milk yield

The multiple trait prediction (MTP) method [6] with the Wood’s model [9] was used to predict the milk yield up to 305 days for each cow using her individual daily milk yield records. Since MTP takes into account the variation of milk yield for each day of milk (*t*) and the variation of curve parameters within each cow group, 305-day milk yield was predicted for each breed group, separately due to differences in average milk yields between Jersey and Jersey-Friesian cows [10].

The following restrictions were made for records to be eli gible for 305-day milk yield prediction. To estimate phenotypic variances for each day in milk across cows, records from at least 300 cows on each day were used. To estimate the curve parameters for each cow, cows with more than 200 daily milk records, with at least one record within the first 20 days in milk and after 300 days in milk were used. For prediction of 305-day yields, only cows with at least 100 daily milk records were used, including the presence of at least one record within the first 30 days and above 100 days in milk. Accordingly, 88% of the daily milk records from cleaned data were used for prediction of 305-day milk yield available for 91% of cows.

#### Realized 305-day milk yield

For the same cows used to predict 305-day milk yield, observed daily milk yields from day five to a maximum of day 305 were summed up for each cow and realized milk yields were obtained. The R programming language was used for data editing and prediction of 305-day milk yields [11].

### Estimation of genetic parameters

Genetic parameters for aggregated 305-day milk yield traits were estimated with a univariate animal model for all cows’ first and second lactation and for each breed group separately. Genetic parameters were also estimated for repeated (daily milk) records applying random regression and repeatability models for all cows’ first lactation, for a dataset, which combined results from all breed groups. Random regression and repeatability models were not applied for cows in second lactation because lactations were still in progress. For comparison with the variance components estimated with random regression, total milk yield for each 30-day interval across the lactation 1 was also fitted in a univariate animal model.

Models included year-season of calving and month of calving as the contemporary group for lactation 1 and 2, respectively. Season was categorized into two classes as dry (from December to April next year) and wet (from May to November) seasons and there were six, year-season effects in total (three years with two seasons). Herd of origin information where they were reared in Australia before importation was not available for these cows. Therefore, all cows were treated as being from the same herd. Cows calved for the first time when they were 578 to 1,493 days of age. Age was not significant and therefore was not included in the model. The minimum size of the contemporary group was maintained as eight to minimize data loss and records from smaller contemporary groups were omitted. For the realized 305-day milk yield trait, lactation length was used as a linear covariate.

#### Univariate animal model for aggregated milk yield traits and for total milk yield in each day in milk class

The predicted and realized 305-day milk yield in lactation 1 and 2, as well as the total milk yield for each 30-day interval across lactation 1, were fitted in a univariate animal model. Analyses of 305-day milk yields were conducted for each lactation separately. The data on daily yields of the first lactation were divided into ten subsets according to days in milk, namely 5–35, 36–65, up to 276–305 days. Each subset was considered as a different trait and total milk yield for each period was calculated. The univariate animal model fitted was as follows:

y=Xb+Za+e

Where ***y*** = vector of observations for aggregated milk yields in lactation 1, lactation 2, or for 30-day periods within lactation 1, ***b*** = vector of fixed effects of year-season (for lactation 1), month of calving (for lactation 2) and lactation length as a covariate (for realized 305-day and 30-day milk intervals), ***a*** = vector of random animal effects, ***e*** = vector of random residual effects, and ***X*** and ***Z*** are design matrices which relate records to fixed effects and random animal effects, respectively.

#### Repeatability model for daily milk records

Daily milk records were used to fit the repeatability model for lactation 1.

y=Xb+Za+Wpe+e

Where ***y*** = vector of daily milk records in lactation 1, ***b*** = vector of fixed effects of year-season and breed, ***a*** = the vector of random additive genetic effects, ***pe*** = vector of random permanent environmental effects and non-additive genetic effects and ***e*** = vector of random residual effects. ***X, Z*** and ***W*** are incidence matrices relating records to the fixed, random animal and permanent environmental effects, respectively.

The variance components for the random effects were de noted as $Var(a)=A\sigma_{a}^{2},Var(\mathrm{pe})=I\sigma_{pe}^{2}$, and $Var(e)=I\sigma_{e}^{2}$, where ***A*** is the additive genetic relationship matrix, $\sigma_{a}^{2}$ is the additive genetic effects variance, ***I*** is the identity matrix, $\sigma_{pe}^{2}$ is the permanent environmental variance and $\sigma_{e}^{2}$ is the residual variance. The covariance among ***a***, ***pe***, and ***e*** were assumed to be zero.

#### Random Regression model for daily milk records

A univariate random regression model was fitted using *k*th order Legendre polynomials for daily milk records in the first lactation. The following model was applied.

y=Xb+Qa+Wpe+e

where ***y*** = vector of daily milk records, ***b*** = vector of fixed regression coefficients, ***X*** = incidence matrices relating to the fixed effects of year-season, breed and a Legendre polynomial for days in milk, ***a*** and ***pe*** are vectors of random Legendre polynomial regressions on days in milk for animal additive genetic and permanent environmental coefficients on days in milk, ***Q*** and ***W*** are corresponding incidence matrices for additive genetic and permanent environmental random effects, respectively, ***e*** is the vector of residual effects. Assume that the distribution of the observations conditional on all model terms other than the residual,

$$y|b,a,\mathrm{pe},\sigma_{e_{1}}^{2}\ldots \sigma_{e_{10}}^{2}\sim N(\mathrm{Xb}+\mathrm{Qa}+\mathrm{Wpe},R),$$

and the covariance structure of the random terms in the model (***V***),

$$\left(\begin{array}{c} a\\ \mathrm{pe}\\ e \end{array}\right)\sim N(0,V)$$

with

$$V=\left[\begin{array}{ccc} G⊗A & 0 & 0\\ 0 & P⊗I & 0\\ 0 & 0 & R \end{array}\right]$$

where ***G*** and ***P*** are *k*×*k* the (co)variance matrices of the random regression coefficients for additive genetic and permanent environmental effects, ***A*** is the additive genetic relationship matrix among animals, ⊗ is the Kronecker product, ***I*** is the identity matrix, ***R*** is diagonal matrix with elements that depend on days in milk i.e., $R=diag\left\{\sigma_{e_{k}}^{2}\right\}$, where k denotes the number from 1 to 10 defined for the range of 30-day intervals starting at day five in milk. Thus the diagonal ***R*** contained 10 different variances related to these periods.

Variance components based on the univariate animal model were estimated for each breed separately with the BESSiE software [12] using a Bayesian approach. A blocked Gibbs sampler was run for 50,000 cycles, with scaled inverted Wishart distributions assigned as prior processes to the residual and additive genetic co-variance matrices with parameter “ν” set to “x” and “y”, respectively (Sorensen and Gianola [13], pages 576 to 588 for further details). The additive genetic ($\sigma_{a}^{2}$) and residual co-variance matrices were calculated as posterior means by averaging the sum of every 100th iteration omitting the first 1,000 iterations as burn-in. The heritabilities were estimated as $\sigma_{a}^{2}/\sigma_{p}^{2}$, where $\sigma_{p}^{2}$ is the sum of additive and residual variances ($\sigma_{a}^{2}+\sigma_{e}^{2}$).

Variance components for repeatability and random regres sion models were estimated using the WOMBAT software [14]. The most appropriate order of fit for each of the random regression model was selected based on the logarithm of the restricted maximum likelihood function (LogL), Akaike’s information criterion (AIC) [15] and Bayesian information criterion (BIC) [16]. The output of WOMBAT presents the AIC and BIC multiplied by −1/2 which makes them similar scale to LogL so that the greatest value corresponds to the best model.

#### Estimation of breeding values from a univariate animal model

Estimated breeding values (EBVs) for 305-day predicted milk yield were obtained using an animal model. The sire EBVs based on the Sri Lankan data were regressed on the sire EBVs from Australia to compare the sire EBVs calculated in two countries. Pearson product-moment correlations between the bull EBVs in Australia and bull EBVs in Sri Lanka were derived from 305-day milk yield for Jersey and Jersey-Friesian cows, separately.

## RESULTS

**Figure 1:**
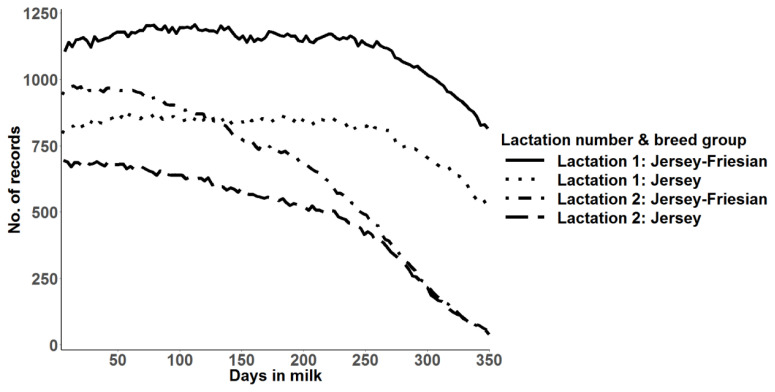
Number of daily milk yield records used for prediction of 305-day milk yield and random regression analysis of daily milk yield for Jersey and for Jersey-Friesian cows in lactation 1 and 2.

Jersey-Friesian cows produced higher mean daily milk yield than Jersey cows, especially in the second lactation (Table 1). The number of daily milk records in the second lactation was lower than in the first lactation, especially towards the end of lactation, as most second lactation cows had not completed their lactation at the time of analysis (Figure 1). After initial data edits, the mean and the range of number of daily milk records per cow in first and second lactations were 280 and 1–342 and 205 and 1–337, respectively.

**Figure 2:**
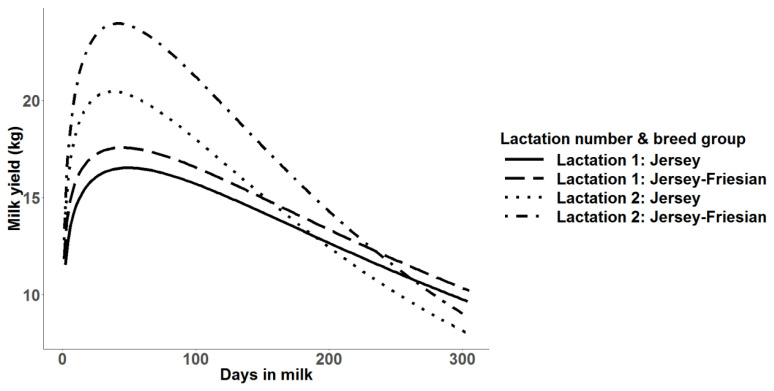
Mean predicted 305-day milk yield with multiple trait prediction for Jersey and Jersey-Friesian cows in lactation 1 and 2.

Similar to mean daily milk yield, higher mean predicted 305-day milk yields were observed in Jersey-Friesian cows than Jersey cows. In the second lactation, mean predicted 305-day milk yield was higher than in the first lactation (Table 2). The coefficient of variation (CV) was highest in the realized 305-day milk yield, followed by predicted 305-day milk yield. The highest CV in realized 305-day milk yield was expected due to the variation in days in milk used for the calculation. The minimum and the maximum number of days used for realized 305-day milk yield calculation ranged from 112 to 299 and from 91 to 299, for Jersey and for Jersey-Friesian cows, respectively. Correlations between realized and predicted 305-day milk yield were 0.86 and 0.88, for Jersey and for Jersey-Friesian cows, respectively. The peak milk production was about 18 kg for Jersey-Friesian cows and 17.5 kg for Jersey cows in first lactation and was observed at about 60 days in milk (Figure 2). The peak milk production in second lactation was higher than first lactation in both Jersey (20 kg) and Jersey-Friesian (24 kg) breed groups. However, after around 150 days, daily milk production in second lactation gradually declined below the first lactation for both breed groups (Figure 2).

### Genetic parameters for milk yield traits

In this study, heritability estimates for predicted and realized 305-day milk yield traits were low (Table 3). The estimate for Jersey-Friesian cows was very low (0.02±0.01), whereas that for Jersey cows was slightly higher (0.08±0.03) in first lactation. The same trends were observed in the second lactation. In both breed groups, the heritability estimates were similar in both lactations.

The heritability estimates of Jersey and Jersey-Friesian cows for predicted and the realized 305-day milk yield were similar (Table 3). Although the phenotypic variance was lower for realized 305-day milk yield compared to the predicted 305-day milk yield, the observed variance (sd^2^, Table 2) was higher in realized 305-day milk yield compared to predicted 305-day milk yield. This lower phenotypic variance for realized milk yield was due to fitting the days in milk as a covariate in realized 305-day milk yield. Variance components for total milk yield in each 30-day interval as estimated by univariate animal model are given in Table 4, and showed low additive genetic variation resulting in low heritability.

#### Repeatability

Additive genetic, permanent environment and residual variances estimated using the repeatability model for daily milk yields for both Jersey and Jersey-Friesian cows were 0.01, 6.30, and 16.50, respectively. Heritability and repeatability estimates were therefore 0.00±0.004 and 0.276± 0.007, respectively.

#### Random regression

**Figure 3:**
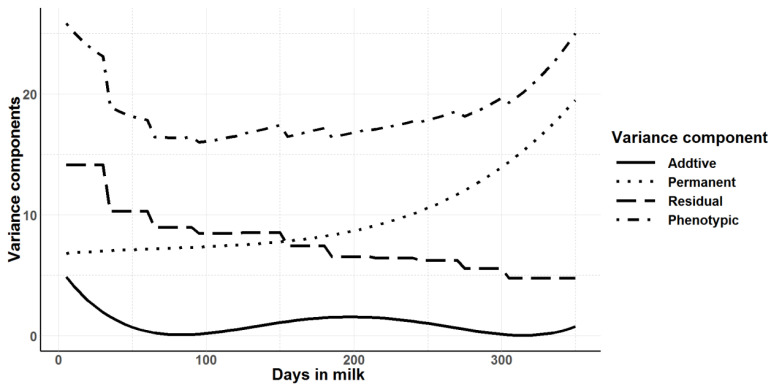
Additive genetic, permanent environment, residual and phenotypic variances for daily milk yields in first lactation from the random regression model with second order Legendre polynomial.

**Figure 4:**
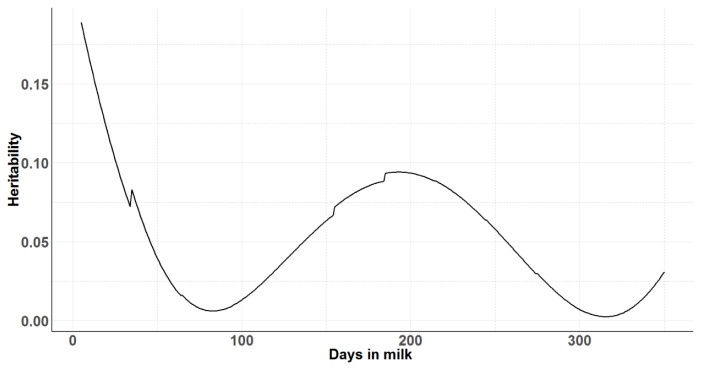
Heritability over days in milk for daily milk yields from the random regression model with second order Legendre polynomial for lactation 1.

Legendre polynomials from order zero (null model) to order 3 for random and permanent environmental effects were evaluated. Random regression models of third order (degree 3) for both additive genetic and permanent environmental across the lactation had significantly higher log-likelihood, −1/2 of AIC and BIC values indicating a better fit of the models. However, with increasing order of Legendre polynomials, fluctuations of curves were observed at the beginning and at the end of lactation (data not shown). Therefore, model with the second order Legendre polynomials for both additive genetic and permanent environmental across the lactation was selected as the best model. The estimated variances along the trajectory for additive genetic, permanent environment, residual and total phenotypic variances estimated using daily milk yields across the first lactation are shown in Figure 3. The shape of the residual variance curve and hence the phenotypic variance along the lactation trajectory shows the heterogeneity of residual variance across the different lactation periods. Similar to the univariate animal model fitted for 305-day milk yield and for each 30-day class along the lactation length, the additive genetic variance observed in the random regression model was very low. In the random regression model, the heritability for daily milk yield ranged from 0.002±0.05 to 0.19±0.02 depending on the stage of lactation (days in milk) (Figure 4). Average heritability across the lactation was 0.05± 0.04 for the random regression model. Heritability was lowest at the peak milk yield and at the end of lactation.

The phenotypic correlations between consecutive days in milk were higher and correlations decreased as the distance between days in milk increased (Table 5). The phenotypic correlations ranged from 0.14±0.02 to 0.71±0.01 with low standard errors. The magnitude of the genetic correlations showed a clear distinction in three phases of lactation namely, 6 to 55 days, 80 days to the days in milk in later lactation and 105 to 255 days in milk. However, no such clear distinction in phenotypic correlations was observed. The genetic correlations were highest between adjacent days in milk between 105 to 280 days with correlations greater than 0.90. Genetic correlations were close to one from 130 to 255 days in milk. Genetic correlations between milk yields at 80 day were positively correlated with days in milk in the later lactation with high standard errors. Yields at 6 to 55 days in milk with consecutive days in milk in the later lactation up to 280 day were negative due to extrapolation of curves at the ends in random regression. The additive genetic variation was close to zero around the peak milk production (Figure 3). Therefore, genetic correlations could not be estimated or yielded high standard errors. The observed genetic correlations between 105 to 280 days in milk indicate that this period can be considered as a single trait.

#### Estimated breeding values

The variance of bull EBVs for 305-day milk yield in Australia and Sri Lanka were 73,532 kg^2^ and 414 kg^2^, respectively. The EBVs calculated for 305-day milk yield in Sri Lanka for Jersey and Jersey-Friesian crossbred cows were regressed on EBVs found for the same Jersey bulls in Australia. The regression equations for Jersey bulls used for Jersey and Jersey-Friesian cows were *y* = 5.229 +0.013*x* (Adjusted *R*^2^ = 8.5%) and *y* = 4.783+−0.015*x* (Adjusted *R*^2^ = 5.7%), respectively, where *y* = sire EBVs in Sri Lanka and *x* = sire EBVs in the Australia. Pearson product-moment correlations between the bull EBVs in Australia and bull EBVs in Sri Lanka for 305-day milk yield were 0.39 in Jersey cows and −0.35 in Jersey-Friesian cows. Therefore, the ranking of sires across the two environments was different as well as difference in EBVs found in Sri Lanka were smaller than Australia.

## DISCUSSION

### Predicted 305-day milk yield

Since the standard period for comparison of milk production yields or genetic parameters is 305-day, milk yields were predicted to 305-day. Although daily milk records increased the density of trajectorial data considerably in this study, the prediction was still necessary to better accommodate cows with missing daily milk records due to loss of identification tags and partial lactations due to ongoing milking and heavy fluctuations in daily milk yields. Realized 305-day milk yields were then compared with the predicted 305-day milk yield.

The milk production of cows in this study was higher than the milk production from studies earlier reported in Sri Lanka. In Sri Lanka, the temperate breeds and their crossbreds were used to be reared in the hill-country, where there is a comfortable climate for exotic breeds (16°C average annual temperature and >200 mm average annual rainfall). As part of a large project of importation of exotic breeds from Australia to Sri Lanka, Jersey and Jersey-Friesian cows were introduced to the low-country farm where the data for this study were collected as a pilot project to assess the suitability of exotic breeds and their crossbreds for production in the low-country of Sri Lanka. Even though the cows in this study were measured in a low-country environment, the first lactation average 305-day milk yield reported in this study was higher than the average milk yield of Jersey cows reported earlier in hill-country of Sri Lanka [17]. In the same study [17], the crossbred cows of exotic breeds (Friesian, Jersey, and Ayrshire) had a similar level of performance to this study. The higher milk production in this study was potentially the result of temperature control through mist in the milking parlour and improved feeding management.

Though the 305-day milk production in this farm has increased in comparison to previous studies in Sri Lanka, the peak milk production and 305-day predicted milk yield observed in this study were substantially lower than the milk yield of Holstein and Jersey cows in the temperate countries [18,19]. In New Zealand, the cows were mostly managed under pasture based management conditions and the average dairy cow milk production (4,259 kg) is similar to Sri Lanka [20]. However, the peak milk production of Holstein-Friesian cows in New Zealand is high (25.5 kg) [21]. In Sri Lanka, lower peak milk production is expected due to heat stress and differences in feed composition and overall management. Hence, there is potential to further upgrade milk production through improved heat regulation and nutritional management with emphasis on economic aspects.

### Low heritability estimates for milk yield

The results of both univariate animal model, as well as random regression models, show that the heritability estimates for milk yield in Jersey and Jersey-Friesian cows in Sri Lanka were low. Generally, exotic cattle breeds reared in tropical climates had lower heritability estimates for milk yield than temperate climates [22]. However, in this study the heritability estimates were much lower than what is usually found for milk yield traits in tropical countries. The heritability for 305-day milk production was lower than Danish Jersey heifers imported to Sri Lanka in 1974 (0.22±0.23) [23], Kenyan Holstein-Friesian (0.25±0.04) [22], Jersey cattle in Kenya (0.21) [24] and for Holstein-Friesian cows in Australia (0.32±0.02) [25]. Low heritability estimates similar to our study were reported in Sahiwal cattle in India (0.07) for first lactation [26] and for crossbreds of Sahiwal, Brown Swiss and Ayrshire breeds in Kenya even after accounting for breed proportions (0.09 to 0.13) [27]. Therefore, the low heritability estimates reported in this study have been also observed in some other studies. Low heritabilities can be due to low additive genetic variance or due to high residual variance. In our study we observed both low additive genetic and high residual variances, both reducing the heritability. Possible reasons for low heritability are discussed below.

Comparison of variance components for milk yield traits with literature values has shown that additive genetic variances in our study were low and a major reason for low heritability. For example, an estimate of the additive genetic and phenotypic variances for 305-day milk yield was 360,274 kg and 1,232,113 kg, respectively in Holstein-Friesian cows reared in large scale intensive farms in Kenya as estimated using a repeatability animal model [22]. A low additive genetic variance (36,864 kg) and a phenotypic variance (164,836 kg) similar to our study were reported in Danish Jersey heifers imported and reared in hill-country of Sri Lanka in 1974 [23]. Another study for first lactation 305-day milk yield in Sahiwal cattle in India, also reported a lower additive genetic variance (64,421 kg) and a phenotypic variance (356,368 kg) similar to our study as estimated by the univariate animal model [28]. The main source of information to estimate the additive genetic variance is the variance of progeny means observed for each breed group for predicted 305-day milk yield. The variance of progeny means is $[(1-t)/n+t]\sigma_{p}^{2}$, where *t* = *h*^2^/4, *n* = number of progeny per sire and $\sigma_{p}^{2}=\text{observed}\;\text{variation}$. For a heritability value of zero, the variance of sire progeny means would be $\sigma_{p}^{2}/n$, and the observed value in our data was close to or even lower than that value implying that there was no extra variation due to sire differences. Small sire differences could be expected if sires were preselected based on EBV. In this study, sires might have selected by targeting bulls with a modest EBV on the assumption that the sires with high EBVs might not fit well to the production systems in Sri Lanka and the sires might even have been selected such that they vary little in EBV. Then the expected production from their daughters will also have a low variation. Such selection was not accounted in the analyses since data on selection was not available. The homogeneity of the population increases with close relationships between sires. In this study, daughters descendent from 39 bulls were used of which parent information for only 22 bulls were available. Among 22 bulls, 12 bulls were half-sibs descending from 4 sires (number of bulls per sire ranged from 2 to 5) and there were 2 bulls that were full-sibs and 3 bulls shared the same sire and maternal grandsire (different dam). Although the model will account for closer relationships and in principle estimate a variance in an unrelated base population, it is possible that some sires were related but unknown to the model in which case the estimated variance will be biased downward. Therefore, low additive genetic variance is a major reason for low heritability in the present study.

The low heritability could also be due to low phenotypic variance since heritability increases with increased level of production [29]. In our study, phenotypic variance and the CV were lower than in other studies [22,30]. A similar phenotypic variance (356,368 kg) to our study was observed in Sahiwal cattle in India with a CV of 0.33 [31]. The differences in phenotypic variance in above studies were mainly due to differences in mean milk production, rearing systems and between sire differences. The higher CV of Kenyan Holstein-Friesian cows (0.35) [22] and Sahiwal cattle in India (0.33) compared to our study (0.17 to 0.26) (Table 2) suggests that differences phenotypic variance could be due to differences in rearing system. The cows in this study were imported to Sri Lanka from Australia as pregnant heifers, and accordingly their rearing environment changed drastically not only by the climatic factors (e.g. heat stress) but also due to nutrition and husbandry levels accompanied with transport stress, all of which could have had significant impacts on expression of cows’ genetic potential and thereby reducing the milk production and phenotypic variance.

There remains a possibility that the low heritability for a moderately heritable milk yield trait could also be caused by pedigree errors. The results of a preliminary study which used part of this dataset reported low heritability estimates for milk electrical conductivity (0.12±0.04), milk flowrate (0.13±0.04), and milk yield persistency (0.07±0.02) [32], and these are similar to the heritability for milking speed (0.11) estimated in a study from the United States [33] and lower than an estimate for milk electrical conductivity (0.23) in Italian Brown cattle [34]. Therefore, the heritability estimates for the above mentioned traits other than the milk yield indicate that the pedigree structure used in this study can provide reasonable estimates of heritability. Low heritability for milk yield estimated in this study is a characteristic of milk yield trait, which is largely influenced by reasons explained above. However, the magnitude of pedigree errors could have been traced accurately if genetic markers were used which were not available for this study.

The heritability estimate could be also low due to errors in phenotype recording. One would expect that with automatic milking systems errors in phenotypic recording would be minimal compared with data measured by humans. However, a study based on data collected by voluntary milking systems in Sweden removed 57% of the original data mainly due to incomplete and inconsistent milk recordings, lactations longer than 330 days in milk and missing identification indicating the need for thorough cleaning of raw data recorded automatically by milking systems [35]. In this study, similar data cleaning criteria were implemented and the heritability estimates remained low for the analysed traits. Therefore, data cleaning criteria were minimized to retain more data. Moreover, high daily fluctuation of milk yield was also observed in the cows in this study. Fluctuations and in particular drops in milk production could be due to diseases and sudden changes in the management [7,36]. However, prediction to 305-day milk yield reduced the effect of the fluctuation in daily milk yield and should give better estimates of variance components and heritability compared to observed milk yield. Therefore, errors in phenotypic recording were not the major reason for low heritability estimates in this study.

Other reasons which might reduce the heritability estimate i.e. increased residual variance was described in detail in this paper under the section “suitability of models used to estimate heritability” together with the variation of the residual, permanent environmental and phenotypic variances along the lactation trajectory. Nevertheless, the incorporation of more data from similar production systems in a future study would strengthen the findings of this study.

### Genetic correlations along the lactation trajectory

Other than the change of variance components along the lactation trajectory, random regression models allow to estimate the genetic correlations between different days in milk. Unexpected negative genetic correlation estimates were observed between the beginnings of the lactation with mid-lactation. The additive genetic variance was close to zero at peak milk production and at the end of lactation at around 305th day. Therefore, the negative genetic correlation estimates could also be due to the difficulty in estimating genetic correlations due to low additive genetic variation at the peak and at the end of lactation. High standard errors of correlation estimates between the consecutive days in milk were also observed, especially from 6 to 80 days in milk with later lactation due to low heritability. Similar negative genetic correlation estimates were observed by Bignardi et al [37].

### Suitability of models used to estimate heritability

Among the univariate, repeatability and random regression models the heritability and additive genetic and phenotypic variances remained low. The univariate animal models for predicted and realized 305-day milk yield were fitted separately for Jersey and Jersey-Friesian cows to visualize the differences in heritability estimates between the two breed groups. The repeatability and random regression models were fitted for both breed groups in a single analysis to enable the modelling of environmental effects with a large data set. These models harbour extra information compared to univariate models.

The repeatability models allow permanent environmental effects to be accounted for repeatability of daily milk records to be estimated. The repeatabilities reported in the literature for milk yield vary from 0.3 [27] to 0.53 [38]. The low repeatability for milk yield reflects a considerable influence of temporary random environmental conditions. Repeatability is important from a management point of view to get similar production from day to day throughout the lactation.

Compared to the repeatability model, random regression models account for the curvilinear nature of lactation and provide more information on the change of variance components along the lactation trajectory. At the beginning of the lactation and towards the end of the lactation, the variance components were increased compared to the mid-lactation. When second order polynomials are fitted, the distribution of predicted animal variances tends to curve at the ends due to artifacts of the Legendre polynomials rather than due to actual increase in variance [39]. This could be due to the presence of fewer records at the beginning and at the end of lactation, which allows more fluctuations of polynomials in the tails. A separate analysis of the total milk yield using the univariate animal model where the milk yield was divided into 10 day periods further confirmed the higher genetic variances observed at the ends of the lactation are due to artifacts of the Legendre polynomials rather than actual increase in variance. A similar trend was observed for additive genetic, permanent environmental effects and residual variances.

Apart from those at the beginning and end of the record ing schedule, the highest additive genetic variance for milk yield was observed at mid-lactation. Though the heritability and additive genetic variance was lower in this study, the trend for additive genetic variance and heritability to peak in mid-lactation was also observed by Druet et al [40], where a maximum heritability of 0.39 was found close to 200 days in milk.

Residual variances were much higher in this study than permanent environmental effects whereas the reverse was reported in most of the other studies [41]. A study by Druet et al [40] reported decreasing residual and permanent environmental variances along the lactation curve in first lactation of French Holsteins. The residual variance could be increased by not accounting for relevant fixed effects or the covariates in the genetic model. For example, herd of origin information is not available for the cows where they were reared in Australia before conception, which was likely to affect the permanent environment of the cows. Due to a lack of such information, herd before conception could not be included in the model, which may have increased the error variance of the model at the beginning of the lactation. The genetic superiority in F1-crossbreds is mostly due to the heterosis and in the model fitting, it was usually captured by the permanent environmental effects [27]. Even though the milk production from Jersey-Friesian cows was higher than Jersey, this could explain why the heritability was low in Jersey-Friesian cows compared to Jersey cows. Moreover, the dam and breed proportions for the Jersey-Friesian cows were not available which may also increase the residual variance. Therefore, this pattern of residual and permanent environmental effects was expected.

### Estimated breeding values and genotype by environment interaction

Variance of EBV observed in this study was very low due to low additive variance. A study in Australia which used genomic relationships between animals and their phenotypes to estimate genetic variance showed variance in EBVs for 305-day milk yield as 41,910 in Jersey cows and 61,332 in Holstein cows [42]. EBVs for milk yield in Sri Lanka were all close to zero due to the low genetic variation which makes it difficult to identify superior bulls for selection based on the data available. Low EBVs also make it difficult to accurately estimate the extent of genotype by environment interaction and interpretation of it. However, genotype by environment interaction is important in this study to compare the bull performances in Australia and Sri Lanka.

In this study, we did not have data from Australia, but Pear son product-moment correlations between the Sri Lankan bull EBVs and Australian bull EBVs indicated a genotype by environment interaction. The environment provided in Sri Lanka could have reduced the proportion of variation attributed to the additive effects of genes, hence reduced the expression of genetic variance in milk production. Therefore, a very slow genetic progress by importing animals, cows or semen from Australia to the low-country of Sri Lanka could be expected.

Genotype by environment interaction was also reported in hill-country of Sri Lanka where there is a comfortable climate for the exotic cows. Accordingly, a similar study based on cow importation from Denmark to a farm in the hill-country in Sri Lanka in 1974 also found a very low additive genetic correlation between the two environments based on the progeny in both environments (−0.08) [23]. Overall these results demonstrate that the genotype by environment interaction have to be carefully considered in any future importations of exotic cows to Sri Lanka.

### Recommendations

Implementation of a breeding programme for dairy cows in Sri Lanka under the intensive system of management requires the recording of pedigree and economically important traits. The price per litre of milk in Sri Lanka is determined by milk fat content and milk solids. Therefore, measuring the individual cow fat and protein yield is also important. The parlour data provide useful information without the need of an extra cost for recording. Therefore, other possible economically important traits, which can be recorded automatically by the parlour, should be incorporated considering the cost of manual recording and the low accuracy of those records. The use of free-roaming bulls in the herd to breed the non-pregnant cows should be avoided at all times since it interferes with the proper pedigree recording. The continued production from imported stock, especially crossbreds depend on the proper selection of bulls for mating and this also emphasizes the need for proper record keeping. Thereby the imported cows and their progeny would serve as foundation stock for within-country evaluation of bulls. Importation of young heifers is preferred over pregnant heifers since transport stress might affect the first lactation milk production. In future, any heifer importations should be on the condition that information describing herd of origin, breed proportions, and complete pedigree including dam and grandparent information, which facilitate the genetic parameter estimation, be available.

Assuming that the imported cows will properly adapt to the Sri Lankan environmental conditions with time, the later lactations of these cows and their daughter performances should be evaluated in a future independent study. Their ability to express genetic potential will help to identify the genetic differences between cows which enable a within-country evaluation of bulls to produce locally adapted dairy cows.

## CONCLUSION

The automatically recorded daily milk records in milking parlours need careful data cleaning and provide a higher data density along the lactation trajectory for each cow. These records were sufficient for a routine genetic evaluation of cows for breeding programmes. Low heritability for 305-day milk yield estimated in this study demonstrate limited potential for genetic improvement of yield based only on data from imported cows in the selected farm and sire differences in Australia were not observed in Sri Lankan data. Therefore, continual phenotype and pedigree recording from the same farm and incorporation of data from other farms with similar production systems is suggested for genetic evaluation to find adequate genetic differences among cows raised in Sri Lanka which enables within-country evaluation of bulls for selection.

## Figures and Tables

**Table 1:** Descriptive statistics of daily milk yields (kg) from day five to day 350 for lactation 1 and 2

Items	No. of cows	No. of records	Mean	Standard deviation	Coefficient of variation	Minimum	Maximum
Lactation 1							
Jersey	991	276,975	13.78	4.41	0.32	2.00	33.42
Jersey×Friesian	1,381	386,915	14.65	4.95	0.34	2.00	33.47
Lactation 2							
Jersey	799	168,201	16.05	6.02	0.37	2.00	44.24
Jersey×Friesian	1,106	222,834	18.94	6.77	0.36	2.00	44.20

**Table 2:** Descriptive statistics of predicted 305-day milk yield (kg) and realized 305-day milk yield (kg) from daily milk yields for lactation 1 and 2

Items	No. of cows	Mean	Standard deviation	Coefficient of variation	Minimum	Maximum
Lactation 1						
Predicted 305-day						
Jersey	900	4,169	707	0.170	2,055	5,961
Jersey×Friesian	1,253	4,416	850	0.192	1,806	6,935
Realized 305-day						
Jersey	900	3,844	809	0.210	1,087	5,903
Jersey×Friesian	1,253	4,043	967	0.239	840	6,385
Lactation 2						
Predicted 305-day						
Jersey	657	4,504	834	0.185	1,672	7,122
Jersey×Friesian	913	5,208	887	0.170	2,073	8,185
Realized 305-day						
Jersey	657	3,856	984	0.255	983	7,318
Jersey×Friesian	913	4,277	1,114	0.260	1,002	7,748

**Table 3:** Heritability estimates with standard errors (h^2^±se), additive genetic ($\sigma_{\text{a}}^{2}$) and phenotypic ($\sigma_{\text{p}}^{2}$) variances from univariate analyses for predicted and observed lactation 305-day milk yield for lactation 1 and 2

Trait		h^2^±se	$\sigma_{a}^{2}$	$\sigma_{p}^{2}$
Lactation 1				
Predicted 305-d	Jersey	0.08±0.03	42,973	500,317
	Jersey×Friesian	0.02±0.01	17,473	701,829
Realized 305-d	Jersey	0.09±0.02	32,142	348,032
	Jersey×Friesian	0.03±0.01	14,050	460,394
Lactation 2				
Predicted 305-d	Jersey	0.07±0.03	49,574	684,417
	Jersey×Friesian	0.02±0.01	16,491	743,977
Realized 305-d	Jersey	0.06±0.02	25,104	428,488
	Jersey×Friesian	0.02±0.01	10,351	453,032

**Table 4:** Heritability estimates with standard errors (h^2^±se), additive genetic ($\sigma_{\text{a}}^{2}$) and phenotypic ($\sigma_{\text{p}}^{2}$) variances from univariate analyses for daily milk yields in lactation 1

Trait	h^2^±se	$\sigma_{\text{a}}^{2}$	$\sigma_{\text{p}}^{2}$
5–35	0.03±0.01	155	5,536
36–65	0.04±0.01	231	6,290
66–95	0.03±0.01	163	6,066
96–125	0.02±0.01	165	6,365
126–155	0.04±0.01	275	6,185
156–185	0.02±0.01	132	6,257
186–215	0.02±0.01	146	6,765
216–245	0.02±0.01	136	7,044
246–275	0.01±0.00	94	6,509
276–305	0.02±0.01	155	6,819

**Table 5:** Estimates of heritability (diagonal), genetic (below diagonal) and phenotypic (above diagonal) correlations between days in milk for first lactation derived from random regression models

DIM	6	30	55	80	105	130	155	180	205	230	255	280	305
6	**0.18±0.02**	0.41±0.02	0.39±0.02	0.35±0.02	0.29±0.02	0.24±0.02	0.21±0.02	0.18±0.02	0.16±0.02	0.15±0.02	0.14±0.02	0.15±0.03	0.16±0.03
30	1.00±0.01	**0.08±0.01**	0.40±0.02	0.37±0.02	0.34±0.02	0.30±0.02	0.28±0.02	0.25±0.02	0.24±0.02	0.22±0.02	0.21±0.02	0.21±0.02	0.21±0.03
55	0.95±0.13	0.98±0.07	**0.03±0.01**	0.43±0.02	0.41±0.02	0.39±0.02	0.37±0.02	0.35±0.02	0.34±0.02	0.32±0.02	0.31±0.02	0.29±0.02	0.28±0.03
80	0.32±0.42	0.40±0.34	0.59±0.26	**0.01±0.02**	0.45±0.01	0.44±0.01	0.44±0.01	0.43±0.01	0.42±0.01	0.40±0.02	0.38±0.02	0.37±0.02	0.34±0.02
105	−0.71±0.55	−0.65±0.70	−0.47±1.08	0.43±0.99	**0.02±0.02**	0.48±0.01	0.49±0.01	0.48±0.01	0.48±0.01	0.47±0.01	0.45±0.01	0.42±0.02	0.39±0.02
130	−0.87±0.29	−0.83±0.40	−0.68±0.74	0.18±0.92	0.97±0.06	**0.04±0.02**	0.52±0.01	0.52±0.01	0.52±0.01	0.51±0.01	0.49±0.01	0.47±0.01	0.43±0.02
155	−0.91±0.18	−0.87±0.28	−0.75±0.59	0.09±0.83	0.94±0.13	1.00±0.01	**0.07±0.02**	0.52±0.01	0.52±0.01	0.51±0.01	0.49±0.01	0.47±0.01	0.43±0.02
180	−0.93±0.13	−0.89±0.21	−0.77±0.50	0.06±0.76	0.92±0.18	0.99±0.03	0.99±0.03	**0.09±0.02**	0.59±0.01	0.58±0.01	0.57±0.01	0.56±0.01	0.53±0.01
205	−0.93±0.09	−0.90±0.17	−0.78±0.43	0.04±0.71	0.92±0.24	0.99±0.06	0.99±0.05	1.00±0.00	**0.09±0.02**	0.62±0.01	0.61±0.01	0.61±0.01	0.58±0.01
230	−0.93±0.06	−0.89±0.12	−0.77±0.37	0.05±0.68	0.92±0.29	0.99±0.09	0.99±0.08	1.00±0.02	1.00±0.01	**0.08±0.02**	0.64±0.01	0.64±0.01	0.63±0.01
255	−0.91±0.14	−0.88±0.12	−0.75±0.29	0.09±0.72	0.94±0.39	1.00±0.17	1.00±0.17	1.00±0.09	1.00±0.06	1.00±0.03	**0.05±0.03**	0.67±0.01	0.67±0.01
280	−0.87±0.52	−0.82±0.45	−0.68±0.36	0.19±1.04	0.97±0.74	1.00±0.57	1.00±0.56	0.99±0.45	0.99±0.37	0.99±0.27	0.99±0.13	**0.02±0.04**	0.71±0.01
305	−0.53±2.48	−0.46±2.10	−0.25±1.38	0.63±4.15	0.97±4.60	0.88±3.96	0.88±3.94	0.81±3.31	0.80±3.03	0.81±2.70	0.83±2.19	0.88±1.26	**0.01±0.05**

DIM, days in milk.
